# Widely rhythmic transcriptome in *Calanus finmarchicus* during the high Arctic summer solstice period

**DOI:** 10.1016/j.isci.2020.101927

**Published:** 2020-12-11

**Authors:** Laura Payton, Lukas Hüppe, Céline Noirot, Claire Hoede, Kim S. Last, David Wilcockson, Elizaveta Ershova, Sophie Valière, Bettina Meyer

**Affiliations:** 1 Institute for Chemistry and Biology of the Marine Environment, Carl von Ossietzky University of Oldenburg, Oldenburg, 26111, Germany; 2 Section Polar Biological Oceanography, Alfred Wegener Institute Helmholtz Centre for Polar and Marine Research, Bremerhaven, 27570, Germany; 3 Helmholtz Institute for Functional Marine Biodiversity (HIFMB) at the University of Oldenburg, Oldenburg, 26111, Germany; 4 Plateforme bio-informatique GenoToul, MIAT, INRAE, UR875 Mathématiques et Informatique Appliquées Toulouse, 31326 Castanet-Tolosan, France; 5 Scottish Association for Marine Science, Oban, Argyll PA37 1QA, UK; 6 Institute of Biological, Environmental, and Rural Sciences, Aberystwyth University, Aberystwyth SY23 3DA, UK; 7 Department for Arctic and Marine Biology, Faculty for Biosciences, Fisheries and Economics, UiT The Arctic University of Norway, Tromsø, 9037, Norway; 8 Shirshov Institute of Oceanology, Russian Academy of Sciences, 36 Nakhimova Avenue, Moscow, 117997, Russian Federation; 9 Plateforme Génomique, INRAE US 1426 GeT-PlaGe, Centre INRAE de Toulouse Occitanie, 24 Chemin de Borde Rouge, Castanet-Tolosan cedex, Auzeville 31326, France

**Keywords:** Microbiology, Systems Biology, Transcriptomics

## Abstract

Solar light/dark cycles and seasonal photoperiods underpin daily and annual rhythms of life on Earth. Yet, the Arctic is characterized by several months of permanent illumination (“midnight sun”). To determine the persistence of 24h rhythms during the midnight sun, we investigated transcriptomic dynamics in the copepod *Calanus finmarchicus* during the summer solstice period in the Arctic, with the lowest diel oscillation and the highest altitude of the sun's position. Here we reveal that in these extreme photic conditions, a widely rhythmic daily transcriptome exists, showing that very weak solar cues are sufficient to entrain organisms. Furthermore, at extremely high latitudes and under sea-ice, gene oscillations become re-organized to include <24h rhythms. Environmental synchronization may therefore be modulated to include non-photic signals (i.e. tidal cycles). The ability of zooplankton to be synchronized by extremely weak diel and potentially tidal cycles, may confer an adaptive temporal reorganization of biological processes at high latitudes.

## Highlights

• Transcriptomic rhythms persist in *C. finmarchicus* during the midnight sun period
• Daily transcripts dominate in ice-free environments and at lower latitude
• Ultradian transcripts are revealed under sea-ice and at higher latitude
• Reorganization of the transcriptome might be an adaptation to under-ice tidal cues

## Graphical Abstract

**Figure:**
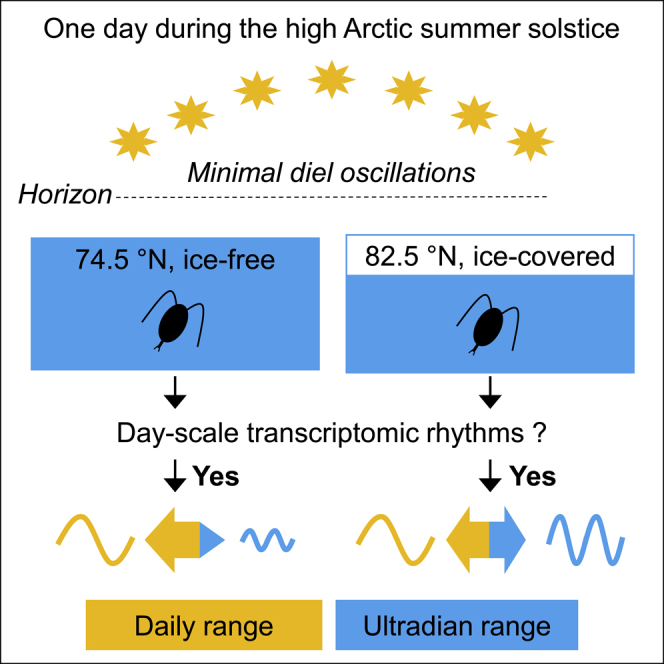

## Introduction

The day/night cycle structures biological processes from gene expression to physiology and behavior (Helm et al., 2017; Mermet et al., 2017). Organisms may respond directly to external stimuli (exogenous) or indirectly (endogenous) via the internal circadian clock. This molecular mechanism enables organisms to track changes in their environment by using the highly predictable light/dark cycle as a *Zeitgeber* (time-giver) although other clocks are known to synchronize to other monotonous cycles (i.e. tidal, lunar, and annual). Endogenous clocks are of adaptive advantage because they enable organisms to anticipate and prepare for predictable environmental changes by temporally organizing short- and long-term biological processes (Helm et al., 2017). However, it is still unclear how this temporal biological organization is facilitated in organisms inhabiting extreme photic environments. In high latitude marine environments without overt day/night cycles such as during the midnight sun period in the Arctic, the sun remains above the horizon for days or months (Abhilash et al., 2017; Bertolini et al., 2019; Bloch et al., 2013; Schmal et al., 2020). Entrainment of the circadian clock by light and associated rhythmic gene oscillations is therefore considered unlikely (Schmal et al., 2020). The persistence of daily rhythms is particularly questionable during the summer solstice, which represents the paroxysmal period of midnight sun, with the lowest diel oscillation and the highest altitude of the sun's position above the horizon (Schmal et al., 2020).

*C. finmarchicus* is a member of the “Calanus Complex”, which constitutes up to 80% of the zooplankton biomass in the Arctic ocean (Søreide et al., 2008). Copepods provide a crucial trophic link between primary production and higher trophic levels, with significant impact on biochemical cycles via the biological carbon pump (Giering et al., 2014; Sanders et al., 2014; Søreide et al., 2008). Moreover, this key planktonic species has been shown to expand its habitat range poleward tracking isotherms as a consequence of climate change (Reygondeau and Beaugrand, 2011). As a consequence it will be exposed to greater annual photoperiodic ranges to which it has evolved (Reygondeau and Beaugrand, 2011), with unknown impacts on its phenology and fitness (Huffeldt, 2020; Saikkonen et al., 2012). A functional circadian clock has been described in this species under clear light/dark cycles (Häfker et al., 2017), and a recent study revealed circadian clock gene transcript oscillation in *C. finmarchicus* in the high Arctic during summer solstice, showing that the core molecular clockwork remains synchronized at this time (Hüppe et al., 2020). It is still unclear whether this clock synchronization leads to daily rhythms.

Copepods are among the important non-model marine invertebrates for which genomic resources are still limited, one barrier being that many species, including *C. finmarchicus*, have large genomes, difficult to sequence (Bron et al., 2011; Choquet et al., 2019; Tarrant et al., 2019a). The *de novo* transcriptome of *C. finmarchicus* (Lenz et al., 2014) has provided a platform to study the expression of rhythmically expressed mRNAs (Hughes et al., 2017; Li et al., 2015a; Mermet et al., 2017). Indeed, at the molecular level, the endogenous clock machinery drives the rhythmic expression of downstream genes whose rhythmic translation and function ultimately underlie daily oscillations at a cellular and organismal level (Li et al., 2015b).

In order to determine the persistence of daily rhythms during the midnight sun, we investigated *in situ* daily transcriptomic rhythms in *C. finmarchicus* during the summer solstice at a southern (74.5° N) sea-ice-free and a northern (82.5° N) sea-ice-covered station in the Barents and Arctic Seas respectively.

## Results and discussion

### Evidence of rhythmic transcriptomes during the summer solstice in the high Arctic

**Figure 1:**
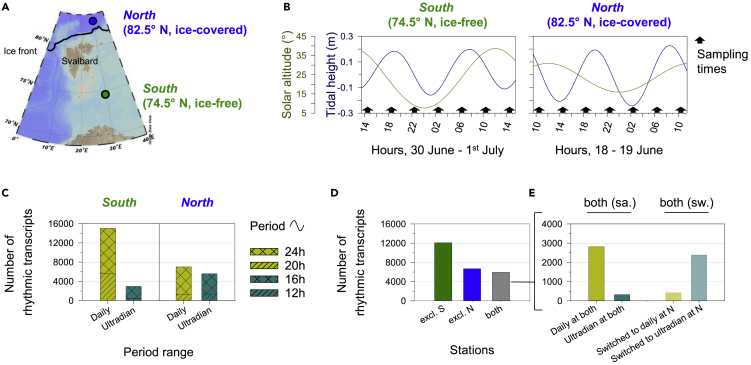
Sampling strategy and results of the rhythmic analysis at *South* (74.5°N, ice-free) and *North* (82.5°N, ice-covered) stations (A) Map with sampled stations *South* (74.5 °N, ice-free) and *North* (82.5 °N, ice-covered) and the position of the ice edge at the day of sampling at *North*. (B) Solar altitude above the horizon (°, dark yellow) and tidal height (m, dark blue) cycles over the sampling times at each station. Sampling of *Calanus finmarchicus* (indicated by black arrows) covered a complete 24h cycle at 4h intervals at each station, from the 30^th^ June at 14-15h to 1^st^ July at 14-15h at *South* (9 days after summer solstice), and from the 18^th^ June at 10-11h to the 19^th^ June at 10-11h at *North* (3 days before summer solstice). For each time point and station, RNA sequencing was performed on 3 pools of 15 CV stage C. *finmarchicus*. The time was indicated in hours, local time (UTC +2). C-D-E. Results of rhythmic transcripts quantification (RAIN algorithm) with an adjusted-p-value cutoff of 0.001. (C) Number of daily (20h and 24h, mustard yellow) and ultradian (12h and 16h, cyan) transcripts at each station. (D) Number of rhythmic transcripts (both daily and ultradian) exclusively at *South* station (“excl. S”), exclusively at *North* station (“excl. N″), and at both stations (“both”). (E) Details on rhythmic transcripts at both stations. On the left, rhythmic transcripts at both stations with the same period range of oscillation (“both [sa.]”): daily at both station (full mustard yellow) and ultradian at both stations (full cyan). On the right, rhythmic transcripts at both station with a switch of period range at *North* (“both [sw.]”): ultradian transcripts at *South* switching to daily at *North* (stripped mustard yellow) and daily transcripts at *South* switching to ultradian at *North* (stripped cyan).

We sampled copepods at 74.5° N (south station, ice-free) and 82.5° N (north station, ice-covered) (hereafter referred to *South* and *North* respectively) on the 30° E longitude, within nine days of the summer solstice (Figure 1A). During the sampling periods, the sun remained above the horizon all day at both stations but still showed diel oscillations of altitude (Figure 1B) and photosynthetic active radiation (Hüppe et al., 2020). *North* experienced lower diel oscillations of the sun's altitude due to the higher latitude and proximity to the summer solstice when compared to *South*. Furthermore *North* was under snow covered sea-ice, attenuating light irradiance and spectral composition of the water column (Wallace et al., 2010). Both stations exhibited semidiurnal tidal cycles (~12.4h), with slightly higher tidal amplitudes at *North*. Net sampling at both stations was performed at 4h intervals over 24 h and the gene expression of *C. finmarchicus* (CV stage) was analyzed on the transcriptomic level for each time point and station using RNA sequencing as described in Payton et al. (2020) (Figure 1B).

Sequencing yielded a depth of 70.4 million reads per sample, optimizing the detection of rhythmic transcripts (Hughes et al., 2017; Li et al., 2015b) in *C. finmarchicus* which has a large genome (Choquet et al., 2019). For each station, temporal expression of 76,550 transcripts was obtained. These data were analyzed for significant periods at 20h and 24h (hereafter termed “daily”) and 12h and 16h (hereafter termed “ultradian”) periods, assuming that they are the results of an endogenous clock regulation or a direct response to environmental factors (Helm et al., 2017) (Table 1).

**Table 1:** Detailed results of the rhythmic analysis (RAIN Algorithm) at *South* (74.5°N, Ice-free) and *North* (82.5°N, ice-covered) stations

Station	Period range	Daily		Ultradian		Daily	Ultradian	All rhythmic
	Period (h)	24	20	16	12			
*South*	**Adj-p < 0.001**	**9 459 (12.4%)**	**5 602 (7.3%)**	**2 533 (3.3%)**	**451 (0.6%)**	**15,061 (19.7%)**	**2 984 (3.9%)**	**18,045 (23.6%)**
	Adj-p < 0.01	18,083 (23.6%)	9 930 (13.0%)	6 327 (8.3%)	1 572 (2.1%)	28,013 (36.6%)	7 899 (10.3%)	35,912 (46.9%)
	Adj-p < 0.05	25,062 (32.7%)	13,168 (17.2%)	10,728 (14.0%)	3 217 (4.2%)	38,230 (49.9%)	13,945 (18.2%)	52,175 (68.2%)
*North*	**Adj-p < 0.001**	**5 754 (7.5%)**	**1 268 (1.7%)**	**4 208 (5.5%)**	**1 404 (1.8%)**	**7 022 (9.2%)**	**5 612 (7.3%)**	**12,634 (16.5%)**
	Adj-p < 0.01	12,864 (16.8%)	2 729 (3.6%)	8 719 (11.4%)	3 993 (5.2%)	15,593 (20.4%)	12,712 (16.6%)	28,305 (37.0%)
	Adj-p < 0.05	20,155 (26.3%)	4 188 (5.5%)	13,620 (17.8%)	7 280 (9.5%)	24,343 (31.8%)	20,900 (27.3%)	45,243 (59.1%)

RAIN results expressed as: number (percentage of total transcripts).

Number of total transcripts = 76,550.

Number (and equivalence in percentage of total transcripts) of transcripts with the most significant results for 24h, 20h, 16h, or 12h period lengths, with an adjusted-p-value cutoff of <0.001, <0.01, and <0.05. Period lengths of 24h and 20h are in the daily period range. Period lengths of 16h and 12h are in the ultradian period range. “All rhythmic” include daily and ultradian transcripts. Results with adjusted-p-value < 0.001 were selected for this study.

Our analysis yielded a total of 18 045 (23.6% of total transcripts) and 12 634 (16.5% of total transcripts) rhythmically expressed genes at *South* and *North* stations respectively (adjusted-p-value < 0.001, Table 1). The number of rhythmic transcripts achieved a total of 52 175 at *South* and 45 243 at *North*, by increasing the adjusted-p-value cutoff to 0.05, representing 68.2% and 59.1% of the total transcriptome respectively (Table 1). Representing the first *in situ* day-scale transcriptomic rhythm analysis in the Arctic Polar region, the results revealed a substantial temporal organization at the transcriptomic level in *C. finmarchicus* during the time of summer solstice, when daily changes in the sun’s altitude are at a minimum, near the lowest anywhere on the planet. A comparable study from the Antarctic Polar region shows that about 600 genes (1.9 % of total transcripts tested) oscillated in Antarctic krill *Euphausia superba* during an Antarctic summer day (Pittà et al., 2013). In the current study, with higher sequencing depth and a more powerful sampling strategy (Hughes et al., 2017; Li et al., 2015b), we show that the number of genes rhythmically transcribed in *C. finmarchicus* in the absence of light/dark cycles is comparable to other marine invertebrates (Biscontin et al., 2019; Connor and Gracey, 2011; Payton et al., 2017; Pittà et al., 2013; Satoh and Terai, 2019; Schnytzer et al., 2018; Tarrant et al., 2019b) or terrestrial mammals (Mermet et al., 2017) in temperate regions.

### Characterization of latitude specific cyclic transcriptome and coincidence with environmental cycles

**Figure 2:**
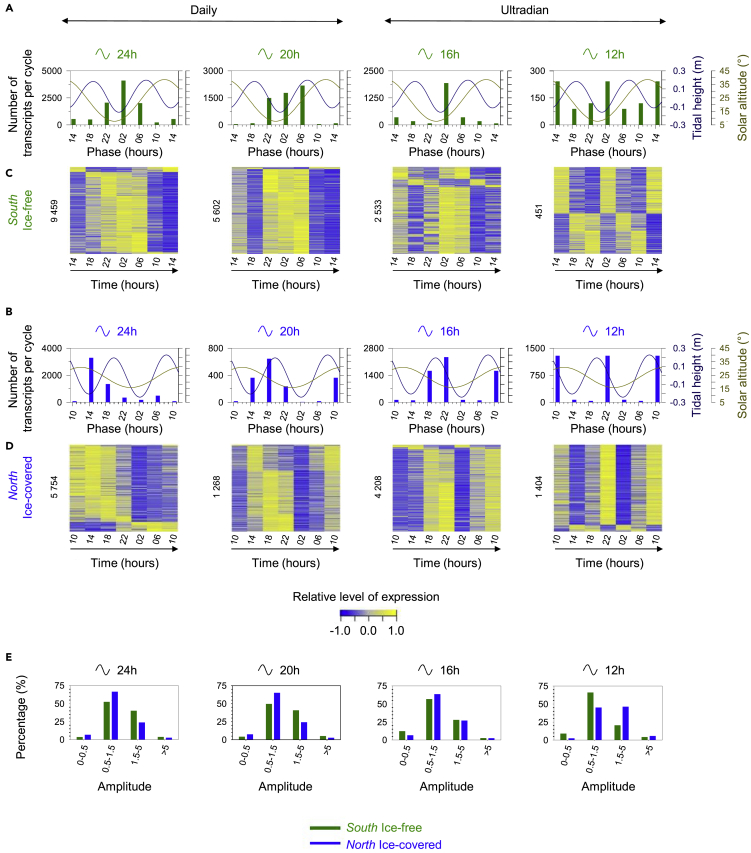
Rhythmic patterns at *South* (74.5°N, ice-free) and *North* (82.5°N, ice-covered) stations and coincidence with environmental cycles (A and B) Phase distributions of daily (24h and 20h period lengths, adj-p < 0.001) and ultradian transcripts (16h and 12h period lengths, adj-p < 0.001) at *South* (A) and *North* (B), according to RAIN algorithm, expressed as the number of transcripts per cycle at each sampling time (hours). Solar altitude above the horizon (°, dark yellow) and tidal height (m, dark blue) cycles over the sampling times at each station were plotted in the background. (C and D). Heatmaps of daily (24h and 20h period lengths, adj-p < 0.001) and ultradian transcripts (16h and 12h period lengths, adj-p < 0.001) at *South* (C) and *North* (D), showing the relative level of expression of rhythmic transcripts along the sampling times (hours), normalized to the median of each transcript. Lowest levels of expression were in blue, highest levels of expression were in yellow, and the transcripts were ordered by phases. The numbers of transcripts for each period length and station were indicated on the left of heatmaps. The time was indicated in hours, local time (UTC +2). (E) Amplitude ranges distribution of daily (24h and 20h period lengths, adj-p < 0.001) and ultradian transcripts (16h and 12h period lengths, adj-p < 0.001) at *South* (green) and *North* (blue), expressed as percentage of rhythmic transcripts per period length and station. An amplitude of 0.5 means that the difference between the minimal and the maximal levels of expression is equal to 0.5 times the minimal level.

Both stations revealed significant daily and ultradian cycling transcripts (adjusted-p-value < 0.001, Table 1 and Figure 1C). This result corroborates with the bimodal oscillations of circadian clock gene transcripts recently described by Hüppe et al. (2020) and validated by the current transcriptomic analysis (Figure S1), characterized by both daily and ultradian rhythms. Interestingly, a lower number of daily transcripts were detected at *North*, where they were 2.1 times less numerous than at *South* (Table 1 and Figure 1C) which may be attributable to lower diel oscillations of solar altitude and the presence of sea-ice cover, and corroborates with the lower amplitude of daily oscillation of *clock, period 1* and *timeless* at *North* (Hüppe et al., 2020). To increase understanding of daily transcripts at both stations, phase of gene expression with that of the environmental cycles was determined, i.e. the time of peaks of expression during the reporting period (Figures 2A–2D). Differences in phase of daily transcripts was observed between *South* (Figures 2A and 2C) and *North* (Figures 2B and 2D). While daily transcripts at *South* could be defined as “nocturnal”, as most peak expressions occurred when solar irradiance reached its daily minimum between 22h and 7h, most of the daily transcripts at *North* peaked between 14h and 23h, when solar altitude and irradiance was decreasing (Figures 2A–2D). A consistent phase shift of expression between stations was observed in the expression of the positive regulators of the circadian clock: *clock* peaking at 6h at *South* and 19h at *North; cycle* peaking at 22h at *South* and 19h at *North* (Hüppe et al., 2020). In addition to being more numerous at *South*, the proportion of daily transcripts with high amplitudes (1.5–5; >5) is greater at *South* than at *North* (Figure 2E).

In contrast to the daily transcripts, an increase of ultradian transcripts was observed at *North*, where they were 1.9 times more numerous than at *South* (Table 1 and Figure 1C). Thus, the rhythmic transcriptomes at *South* and *North* were characterized by different daily/ultradian ratios: 83.5% were daily and 16.5% were ultradian at *South*, while 55.6% were daily and 44.4% were ultradian at *North* (Table 1 and Figure 1C). These ratios reflect the pattern of circadian clock gene transcripts, for which an increase of ultradian oscillations was clearly observed at *North* (Hüppe et al., 2020). Ultradian transcriptomic rhythms have been increasingly reported over the past decade in a wide range of species (Ananthasubramaniam et al., 2018; Biscontin et al., 2019; Connor and Gracey, 2011; Hughes et al., 2009; Payton et al., 2017; Pittà et al., 2013; Satoh and Terai, 2019; Schnytzer et al., 2018; Tarrant et al., 2019b; Westermark and Herzel, 2013; Zhu et al., 2018). These oscillations often cycle at different harmonics of the circadian rhythm, and among these, the ~12h oscillation is most prevalent (Ananthasubramaniam et al., 2018; Hughes et al., 2009; Westermark and Herzel, 2013; Zhu et al., 2018). Further, ultradian transcriptomic oscillations of ~12.4h, also called (circa)tidal oscillations, are observed in marine organisms under the influence of semidiurnal tidal cycles (Connor and Gracey, 2011; Mat et al., 2020; Satoh and Terai, 2019; Schnytzer et al., 2018). Here, ultradian transcripts phased to tides at both *South* and *North*, with two different phase patterns depending on the station (Figures 2A–2D). At *South*, most of the ultradian transcripts showed a peak of expression with low tides (Figures 2A and 2C), while at *North*, most of the ultradian transcripts showed a peak of expression with high tides (Figures 2B and 2C). In contrast to circadian transcripts, the proportion of ultradian transcripts with high amplitudes (1.5–5; >5) is equivalent to (16h transcripts) or greater (12h transcripts) at *North* than at *South* (Figure 2E).

To further compare *South* with *North*, we analyzed if the same genes were rhythmically transcribed at both stations. The results showed that a large proportion of the total rhythmic transcripts (adjusted-p-value < 0.001) were specific to each station (67.1% at *South*; 52.9% at *North*), with 12 101 transcripts being exclusively rhythmic at *South* and 6 690 exclusively rhythmic at *North* (Figure 1D). These station-specific rhythmic transcripts might reflect differences between ice-free (*South*) and ice-covered (*North*) ecosystems, leading to differential physiological requirements. For example, in ice-covered areas where phytoplankton in the water column is scarce, the sea-ice algae community is a critical food source for copepods (David et al., 2015; Søreide et al., 2008; 2013; Wallace et al., 2010), the nutritional quality of which differs to phytoplankton blooms in ice-free waters (Falk-Petersen et al., 1998). In contrast, the 5 944 transcripts that were rhythmic in animals sampled at both stations, could reflect common physiological requirements (Figure 1D). Of these genes, about half showed distinct changes in their period (Figure 1E) with most (2 388) changing from daily at *South* to ultradian at *North*. We propose therefore, that the pattern of temporal regulation of common physiological processes is specific to each environment and that gene expression is re-organized according to an under-ice habitat.

### Rhythmic biological processes of interest

**Figure 3:**
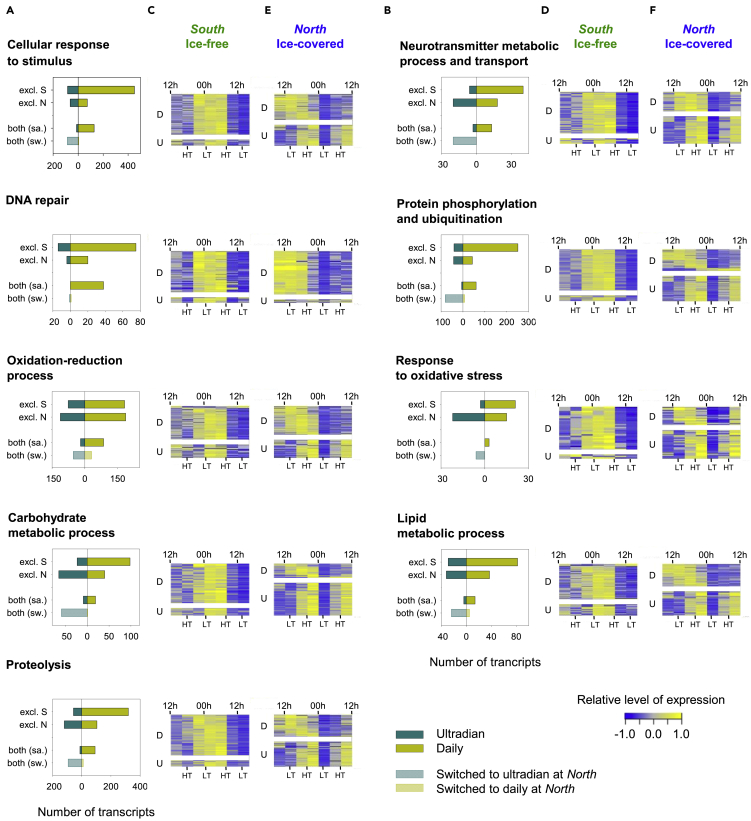
Rhythmic biological processes of interest cellular response to stimulus (GO:0051716), neurotransmitter metabolic process and transport (GO:0042133, GO:0006836), DNA repair (GO:0006281), protein phosphorylation and ubiquitination (GO:0006468, GO:0016567), oxidation-reduction process (GO:0055114), response to oxidative stress (GO:0006979), carbohydrate metabolic process (GO:0005975), lipid metabolic process (GO:0006629) and proteolysis (GO:0006508). (A and B) Details of the rhythmic analysis per biological process. For each biological process, the number of transcripts “excl. S” (transcripts exclusively rhythmic at *South*), “excl. N” (transcripts exclusively rhythmic at *North*), “both (sa.)” (transcripts rhythmic at both stations, with the same period range) and “both (sw.)” (transcripts rhythmic at both stations, with a switch of period range at *North*)” is detailed. For each category, the ultradian transcripts were shown in cyan (full or striped) and the daily transcripts were shown in mustard yellow (full or striped). “both (sw.)” transcripts were shown as expressed at *North*. (C–F) Heatmaps of all rhythmic transcripts per biological process at *South* (C and D) and at *North* (E and F). The level of expression of each transcripts was normalized to the median and transcripts were ordered by phases. Lowest levels of expression were in blue, highest levels of expression were in yellow. The daily transcripts “D” were at the top and the ultradian transcripts “U” at the bottom. The time was indicated in hours, local time (UTC +2). 12h corresponded to the highest and 00h to the lowest solar altitude of the day at each station, “HT”: high tide”, “LT”: low tide. See also Tables S1 and S2.

**Figure 4:**
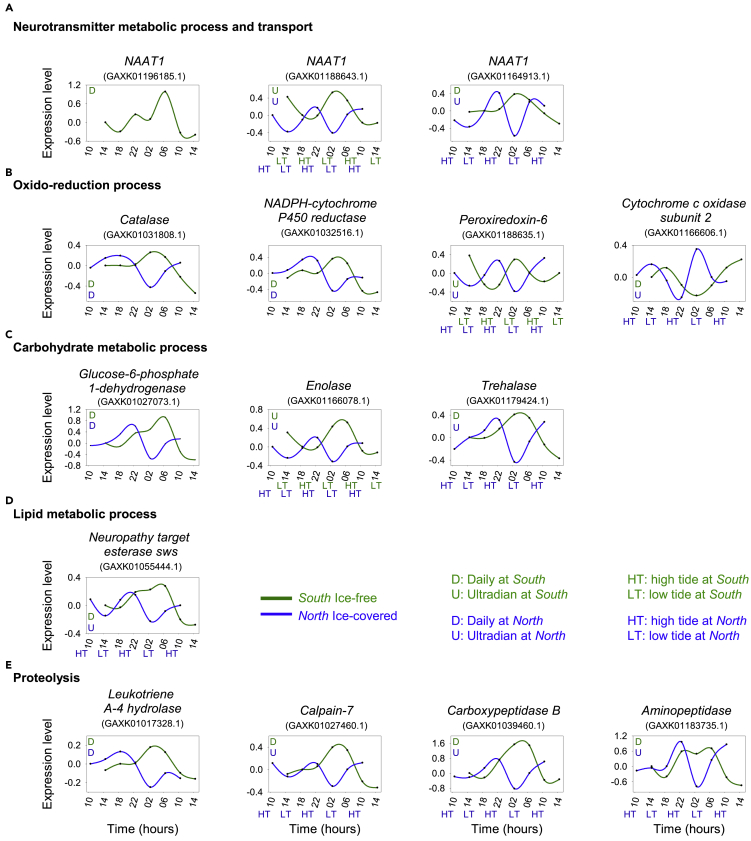
Examples of rhythmic transcripts involved in key biological processes Expression profiles of genes involved in neurotransmitter metabolic process and transport (GO:0042133, GO:0006836) (A), oxidation-reduction process (GO:0055114) (B), carbohydrate metabolic process (GO:0005975) (C), lipid metabolic process (GO:0006629) (D) and proteolysis (GO:0006508) (E) at the *South* ice-free station (green) and the *North* ice-covered station (blue). “D” and “U” correspond to significant daily or ultradian rhythm (adj-p < 0.001), the color corresponding to the station. “HT”: high tide, “LT”: low tide, the color corresponding to the station. Details about rhythmic transcripts presented in this Figure are available Table S2.

Gene ontology (GO) analysis revealed that rhythmic transcripts at both latitudes were particularly common to metabolic and cellular process, signaling, response to stimuli, localization or biological regulation (Figures S2A, S3A, and S4A). To gain a better understanding of the rhythmic biology in *C. finmarchicus*, nine key biological processes particularly observed in rhythmic transcripts (Table S1) were selected based on enrichment analysis (results are presented Figures S2B, S3B, and S4B) to get a more detailed insight into their temporal regulation (Figure 3). Examples of genes from functional groups presented in Figure 3 are provided in Table S2 and Figure 4. By analyzing the rhythmic status of genes involved in these nine key biological processes (Figure 3), we noted that: (1) a combination of daily and ultradian transcripts were observed for each process at both *South* and *North*, rather than daily- and ultradian-specific processes; (2) an increase of ultradian oscillations across all biological processes examined, except “DNA repair”, occurred at *North*, explained by both station-specific and common rhythmic transcripts switching from daily at *South* to ultradian at *North* and; (3) the time of peaks of expression (phases) according to daily or tidal cycles is specific to each station.

Rhythmic patterns of “cellular response to stimulus” supported the observation of the persistence of a daily rhythmic environmental stimulus at both stations, despite the sun always staying above the horizon. Interestingly, over the common rhythmic transcripts between both stations, 128 present daily oscillations at both stations (Figure 3A, both (sa.), full mustard yellow), while 87 switch from daily oscillations at *South* to ultradian ones at *North* (Figure 3A, both (sw.), striped cyan). Finally, while 87% of rhythmic transcripts associated to “cellular response to stimulus” have daily oscillations at *South*, with peaks of expression between 22h and 7h (Figure 3C), the proportion of daily transcripts decrease to 56% at *North*, peaking between 10h and 23h (Figure 3E). In parallel, the proportion of ultradian transcripts increases from 13% at *South* (Figure 3C) to 44% in the *North* (Figure 3E), with clear peaks of expression around high tides at this station (*North*) (Figure 3E). These results support the idea of the increasing response to an ultradian environmental stimulus (e.g. tides) at *North*. This trend is even more accentuated in the rhythmic transcription of genes involved in “neurotransmitter metabolic process and transport” (Figures 3B, 3D, and 3F), for which the proportion of ultradian transcripts is more evident than the one of daily transcripts at *North*. Indeed, the daily/ultradian ratio differs from 89%/11% at *South* (Figures 3D) to 42%/58% at *North* (Figure 3F). Neurotransmitters are involved in a wide range of processes comprising temporal organization, such as photic entrainment of the circadian clock, or transmission of clock outputs such as the circadian food anticipatory activity (Golombek and Rosenstein, 2010; Gotow and Nishi, 2008; Patton and Mistlberger, 2013). Among rhythmic transcripts involved in “neurotransmitter metabolic process and transport” in this study, 3 isoforms of *Sodium-dependent nutrient amino acid transporter 1* (*NAAT1*) are identified (Figures 4A and Table S2B). This amino acid/sodium cotransporter that promotes absorption of essential amino acids has been shown to be expressed with a daily rhythm in circadian neurons of *Drosophila* (Abruzzi et al., 2017). In our study, one isoform of *NAAT1* is rhythmically expressed exclusively at *South,* with a daily rhythm and a peak of expression at 6-7h (Figure 4A and Table S2B). A second isoform shows ultradian oscillations at both latitudes, but showing a phase shift, the peak of expression being at rising tides at *South* and around high tides at *North* (Figure 4A and Table S2B). Finally, a third isoform of *NAAT1* is also rhythmically expressed at both latitudes, but with a daily rhythm at *South* and an ultradian rhythm at *North* (Figure 4A and Table S2B). This result clearly illustrates the environment-dependent modulation of gene expression in terms of period and phase of rhythmic expression. In contrast, the “DNA repair” function, widely described to be under the control of the circadian clock in other species (Borgs et al., 2009), showed the majority of daily oscillations at both *South* and *North*. This result suggests that the increase of ultradian oscillations at *North* may not be beneficial for all functions. However, despite the general common daily pattern for this function, a clear phase shift is noted between stations, with peaks of expression between 22h and 7h at *South* (Figure 3C), and between 10h and 23h at *North* (Figure 3E), highlighting again a clear environment-specific modulation of cellular processes. While chronobiological data are still scarce in marine organisms, some DNA repair genes have been identified to peak during nighttime in the mussel *Mytilus californiaunus* under temperate region’s natural environment simulation in the laboratory (Connor and Gracey, 2020). Another function strongly represented by rhythmic transcripts is “protein phosphorylation and ubiquitination”. The role of posttranscriptional mechanisms in rhythmic regulatory processes is increasingly demonstrated (Mauvoisin et al., 2015; Mermet et al., 2017). Thus, rhythmic transcripts involved in “protein phosphorylation and ubiquitination” suggests continued cyclic regulation at the proteomic level, with again a clear increase of the proportion of ultradian patterns at *North*. We also show clear rhythms in “oxidation-reduction process” and “response to oxidative stress” at both stations. The redox status of organisms involved in many cellular reactions, from respiration to metabolism, appears to be widely rhythmic in all species and all these functions are related to the endogenous clock (Biscontin et al., 2019; Eckel-Mahan and Sassone-Corsi, 2009; O’Neill et al., 2015; Pittà et al., 2013; Putker and O’Neill, 2016). While daily oscillations of redox markers are observed in terrestrial organisms, tidal oscillations, such as *cytochrome oxidase subunits* expression, have also been observed in marine organisms such as the crustacean *Eurydice pulchra* (O’Neill et al., 2015). In the current study, the increase of ultradian regulation at *North* can be illustrated by the switch of the rhythmic expression of *cytochrome c oxidase subunit 2,* from daily at *South* to ultradian at *North* (Figure 4B and Table S2E). However, ultradian oscillations are not limited to the *North* station, as illustrated by the ultradian expression of an isoform of *Peroxiredoxin-6* at both stations, peaking just after low tides at *South*, and just after high tides at *North*. Oxidation-reduction cycles of peroxiredoxin proteins have been thought to constitute a universal marker for circadian rhythms in all domains of life (Edgar et al., 2012). While daily transcription of *peroxiredoxins* have been shown in the Antarctic krill *Euphausia superba* (Pittà et al., 2013), overoxidation of peroxiredoxin follows a circatidal pattern in *E. pulchra* (O’Neill et al., 2015). On the other hand, one of the isoforms of *Catalase*, an important antioxidant enzyme (Nandi et al., 2019), or *NADPH-cytochrome P450 reductase*, an essential component for the function of many enzymes including cytochrome P450 (Weng et al., 2005), exhibit a daily rhythm at both stations, revealing the intertwining of daily and ultradian rhythms of oxidation-reduction processes observed in this study. Finally, we highlighted the temporal expression of transcripts involved in key metabolic processes for energy use and storage in the active CV stage copepodites (Häfker et al., 2018): i.e “carbohydrate metabolic process”, “lipid metabolic process” and “proteolysis” (Figure 3). Most of the common rhythmic transcripts (both [sa.] and both [sw.]) associated to these key metabolic functions switch from daily oscillations at *South* to ultradian ones at *North* (Figures 3A and 3B). Moreover, there is a large proportion of ultradian transcripts in genes exclusively rhythmic at *North* (excl. N), while the daily transcripts are in the majority in genes exclusively rhythmic at *South* (excl. S) for these functions (Figures 3A and 3B). A clear change of the daily/ultradian ratio for rhythmic transcripts associated to these key metabolic processes is observed between *South* (Figures 3C and 3D) and *North* (Figure 3E and 3F). Indeed, while daily transcripts are in the majority at *South*, representing 84%, 76%, and 86% of rhythmic genes involved in “carbohydrate metabolic process”, “lipid metabolic process”, and “proteolysis”, respectively (Figure 3C and 3D); the proportion is much more nuanced at *North*, where the ultradian transcripts even become the majority, representing respectively 69%, 52%, and 52% of rhythmic transcripts at this station (Figure 3E and 3F). These results indicate day-scale oscillations in energetic demands and nutrient supply, with clear modifications of period and phase of oscillations according to stations (Figures 3C, 3D, 3E, and 3F). For instance, isoforms of *Glucose-6-phosphate-1-dehydrogenase* (carbohydrate metabolic process, Figure 4C and Table S2G)*,* identified to be under the control of the circadian clock in *Drosophila* (McDonald and Rosbash, 2001), and *Leukotriene A-4 hydrolase* (proteolysis, Figure 4E and Table S2I), oscillate with a daily pattern at both stations, with clear phase shifts (peaking respectively at 6-7h and 2-3h at *South* and at 22-23h and 18-19h at *North*). In contrast *enolase* transcription is ultradian at both stations (Figure 4C and Table S2G), with again a clear phase shift (peaking after low tides at *South* and at high tides at *North*). This gene encodes for a protein involved in glycolysis and has been shown to have a peak of expression during the night in the Antarctic krill in the lab under L/D exposure (no tides), corresponding to the high level of activity and oxygen consumption in this species in the field (Biscontin et al., 2019). Finally, isoforms of *Trehalase* (Figure 4C and Table S2G), *Calpain-7, Carboxypeptidase B* and *Aminopeptidase* (Figure 4E and Table S2I), involved in carbohydrate metabolic processes and proteolysis, give a clear illustration of the switch of period range according to stations, from daily oscillations at *South* to ultradian ones at *North*. In summary our results highlight plasticity of the rhythmic transcriptome in *C. finmarchicus* suggesting that the widely rhythmic transcriptome is tuned to the cyclic environmental conditions of the prevailing habitat.

### *Persistent cycling of C. finmarchicus* transcriptome during midnight sun

In high latitude marine environments without overt day/night cycles, entrainment of the circadian clock by light and associated rhythmic gene oscillations is considered unlikely (Bertolini et al., 2019; Schmal et al., 2020). However, even during months of permanent darkness or illumination, Polar marine regions remain rhythmic environments, with a persistence of the sun's oscillations below or above the horizon (Cohen et al., 2020; Hobbs et al., 2018; Wallace et al., 2010). Here, the widely daily rhythmic gene oscillations observed at two high Arctic latitude stations during summer solstice, illustrate that subtle daily changes of light intensity or quality are sufficient to synchronize daily molecular rhythms in the key zooplanktonic species *C. finmarchicus*, which is consistent with the high levels of visual acuity recorded in this species (Båtnes et al., 2015).

Some studies show that several Arctic species exhibit daily activity rhythms in the absence of diel light cycles, while others become arrhythmic proposing that the absence of rhythms could be beneficial in polar environments (Abhilash et al., 2017; Bertolini et al., 2019; Bloch et al., 2013). In zooplankton, the most described daily rhythm is the behavior of diel vertical migration (DVM) to the surface at night in order to balance the need to feed close to the surface against the accompanying risk of predation by visually hunting predators (Häfker et al., 2017). DVM has been frequently observed during autumn and spring in the high Arctic when the diel light/dark cycle is present (Dale and Kaartvedt, 2000; Fortier et al., 2001). However, data for synchronized DVM during the Arctic midnight sun are contrasting (Blachowiak-Samolyk et al., 2006; Cottier et al., 2006; Dale and Kaartvedt, 2000; Fortier et al., 2001; Wallace et al., 2010). While the persistence and the purpose of maintaining DVM during this period is under debate, the multitude of daily transcripts observed in this study, including those involved in circadian clock machinery, carbohydrate/lipid metabolism, and proteolysis, suggests that a daily temporal organization at the transcriptomic level could be an advantage for copepods, whether ex- or intrinsic (Abhilash et al., 2017).

However at extremely high latitudes and under sea-ice, gene oscillations become re-organized to include <24 h (ultradian) gene cycles. Entrainment of the circadian (or other) clock(s) and clock-controlled genes may therefore be modulated to include other, non-photic signals (i.e. tidal cycles) (Connor and Gracey, 2011; Mat et al., 2020; Satoh and Terai, 2019; Schnytzer et al., 2018). Interestingly, some genes belonging to eukaryotic translation initiation factors and heat shock proteins are shown to be ultradian in this study, while these genes families have been shown to present conserved harmonic oscillations (ultradian rhythms generated by the circadian clock) between fungi and mammals (Ananthasubramaniam et al., 2018) (Table S3). Here, we propose that ultradian rhythms in *C. fimarchicus* may be entrained by ambient tidal cues such as potential current reversal, food availability, turbulence, salinity or temperature cycles caused by the tides (Massicotte et al., 2020; Oziel et al., 2019; Tessmar-Raible et al., 2011). While ultradian oscillations are also observed at the *South* sea-ice-free station, under-ice currents at *North* station could lead to important tidal cycles of food availability (from ice algae), salinity or temperature (Massicotte et al., 2020; Oziel et al., 2019). Thus, the tidal reorganization at *North* may facilitate for example ingestion rate (Conover et al., 1986; Ibáñez-Tejero et al., 2018; Petrusevich et al., 2020; Schmitt et al., 2011), as suggested by the increase of ultradian oscillations of key metabolic processes in copepods at this station.

The bimodal aspect of the *C. finmarchicus* transcriptomes, presenting both daily and ultradian oscillations, as well as a differential daily / ultradian ratio between stations, were in accordance with the bimodal oscillations of circadian clock transcripts (Figure S1, Hüppe et al., 2020). This corroborated the hypothesis of Hüppe et al. (2020) that the circadian clock could be functional during summer solstice at high latitudes and, as proposed in other species (Enright, 1976; Tran et al., 2020), could be synchronized by both daily and tidal environmental cycles, with a balance between one or the other depending on their relative importance and the associated advantages to synchronize biological processes accordingly, relevant to each ecosystem. Moreover, the station-specific phases of daily and ultradian transcripts compliment the idea of the ability to adapt to site-specific changes of risks and opportunities related to the daily and tidal environmental cycles. Thus, the observed plasticity of rhythmic transcriptomes could be of high adaptive advantage to deal with the specificity of each habitat, and could allow *C. finmarchicus* to adapt to the high Arctic environmental cycles, unrestrained by photoperiod (Huffeldt, 2020; Reygondeau and Beaugrand, 2011; Saikkonen et al., 2012). Finally, daily and ultradian oscillations of key metabolic processes strongly suggest the persistence of feeding and respiration rhythms during midnight sun, with potentially important ecological consequences regarding trophic interactions and biogeochemical processes (Giering et al., 2014; Sanders et al., 2014).

### Limitations of the study

As discussed above, in a context of field study, organisms are exposed to environmental cycles. Thus we cannot rule out that the observed rhythmicity stems from a direct response to light, rather than a clock-controlled regulation. However the consistency between the circadian clock genes expression and the transcriptomic patterns highly suggests a functional clock. Moreover, the endogenous clock(s) controls different layers of regulation to provide robust timing cues at the cellular and tissue level. Here we identified temporal patterns in periodic gene expression by measuring mRNA accumulation. However, the temporal regulation is a dynamic process, including regulation of posttranscriptional mechanisms such as translational efficiency or protein accumulation (Mermet et al., 2017). Thus, further studies at the proteomic or physiological levels are necessary to decipher the exact timing of key biological processes mentioned in this study.

### Resource availability

#### Lead contact

Further information and requests for resources and reagents should be directed to and will be fulfilled by the lead contact, Laura Payton (laura.payton@uni-oldenburg.de).

#### Materials availability

This study did not generate new unique reagents.

#### Data and code availability

The transcriptomic data sets generated during this study are available in the NCBI Bioproject PRJNA628886 (https://www.ncbi.nlm.nih.gov/bioproject/PRJNA628886) and in the figshare collection 5127704 (https://doi.org/10.6084/m9.figshare.c.5127704). All the scripts supporting the current study are available from the corresponding author on request.

## Methods

All methods can be found in the accompanying Transparent methods supplemental file.

## Supplemental information

Document S1. Transparent Methods and Figures S1–S4

Table S1. Details on numbers of transcripts implied in biological processes of interest, related to Figure 3

Table S2. Examples of transcripts involved in rhythmic biological processes of interest, related to Figures 3 and 4

Table S3. Conserved ultradian genes in mammals, fungi, and copepods, related to Figure 4

## References

[bib1] Abhilash L., Shindey R., Sharma V.K. (2017). To be or not to be rhythmic? A review of studies on organisms inhabiting constant environments. Biol. Rhythm Res..

[bib2] Abruzzi K.C., Zadina A., Luo W., Wiyanto E., Rahman R., Guo F., Shafer O., Rosbash M. (2017). RNA-seq analysis of *Drosophila* clock and non-clock neurons reveals neuron-specific cycling and novel candidate neuropeptides. PLoS Genet..

[bib3] Ananthasubramaniam B., Diernfellner A., Brunner M., Herzel H. (2018). Ultradian rhythms in the transcriptome of *Neurospora crassa*. iScience.

[bib4] Båtnes A.S., Miljeteig C., Berge J., Greenacre M., Johnsen G. (2015). Quantifying the light sensitivity of *Calanus spp.* during the polar night: potential for orchestrated migrations conducted by ambient light from the sun, moon, or aurora borealis?. Polar Biol..

[bib5] Bertolini E., Schubert F.K., Zanini D., Sehadová H., Helfrich-Förster C., Menegazzi P. (2019). Life at high latitudes does not require circadian behavioral rhythmicity under constant darkness. Curr. Biol..

[bib6] Biscontin A., Martini P., Costa R., Kramer A., Meyer B., Kawaguchi S., Teschke M., Pittà C.D. (2019). Analysis of the circadian transcriptome of the Antarctic krill *Euphausia superba*. Sci. Rep..

[bib7] Blachowiak-Samolyk K., Kwasniewski S., Richardson K., Dmoch K., Hansen E., Hop H., Falk-Petersen S., Mouritsen L.T. (2006). Arctic zooplankton do not perform diel vertical migration (DVM) during periods of midnight sun. Mar. Ecol. Prog. Ser..

[bib8] Bloch G., Barnes B.M., Gerkema M.P., Helm B. (2013). Animal activity around the clock with no overt circadian rhythms: patterns, mechanisms and adaptive value. Proc. Biol. Sci..

[bib9] Borgs L., Beukelaers P., Vandenbosch R., Belachew S., Nguyen L., Malgrange B. (2009). Cell “circadian” cycle: new role for mammalian core clock genes. Cell Cycle.

[bib10] Bron J.E., Frisch D., Goetze E., Johnson S.C., Lee C.E., Wyngaard G.A. (2011). Observing copepods through a genomic lens. Front. Zool..

[bib11] Choquet M., Smolina I., Dhanasiri A.K.S., Blanco-Bercial L., Kopp M., Jueterbock A., Sundaram A.Y.M., Hoarau G. (2019). Towards population genomics in non-model species with large genomes: a case study of the marine zooplankton *Calanus finmarchicus*. R. Soc. Open Sci..

[bib12] Cohen J.H., Berge J., Moline M.A., Johnsen G., Zolich A.P., Berge J., Johnsen G., Cohen J.H. (2020). Light in the polar night. POLAR NIGHT Marine Ecology: Life and Light in the Dead of Night.

[bib13] Connor K., Gracey A.Y. (2020). Cycles of heat and aerial-exposure induce changes in the transcriptome related to cell regulation and metabolism in *Mytilus californianus*. Mar. Biol..

[bib14] Connor K.M., Gracey A.Y. (2011). Circadian cycles are the dominant transcriptional rhythm in the intertidal mussel *Mytilus californianus*. Proc. Natl. Acad. Sci. U S A.

[bib15] Conover R.J., Herman A.W., Prinsenberg S.J., Harris L.R. (1986). Distribution of and feeding by the copepod *Pseudocalanus* under fast ice during the Arctic spring. Science.

[bib16] Cottier F.R., Tarling G.A., Wold A., Falk-Petersen S. (2006). Unsynchronised and synchronised vertical migration of zooplankton in a high Arctic fjord. Limnol. Oceanogr..

[bib17] Dale T., Kaartvedt S. (2000). Diel patterns in stage-specific vertical migration of *Calanus finmarchicus* in habitats with midnight sun. ICES J. Mar. Sci..

[bib18] David C., Lange B., Rabe B., Flores H. (2015). Community structure of under-ice fauna in the Eurasian central Arctic Ocean in relation to environmental properties of sea-ice habitats. Mar. Ecol. Prog. Ser..

[bib19] Eckel-Mahan K., Sassone-Corsi P. (2009). Metabolism control by the circadian clock and vice versa. Nat. Struct. Mol. Biol..

[bib20] Edgar R.S., Green E.W., Zhao Y., van Ooijen G., Olmedo M., Qin X., Xu Y., Pan M., Valekunja U.K., Feeney K.A. (2012). Peroxiredoxins are conserved markers of circadian rhythms. Nature.

[bib21] Enright J.T. (1976). Plasticity in an isopod’s clockworks: Shaking shapes form and affects phase and frequency. J. Comp. Physiol..

[bib22] Falk-Petersen S., Sargent J.R., Henderson J., Hegseth E.N., Hop H., Okolodkov Y.B. (1998). Lipids and fatty acids in ice algae and phytoplankton from the marginal ice zone in the Barents sea. Polar Biol..

[bib23] Fortier M., Fortier L., Hattori H., Saito H., Legendre L. (2001). Visual predators and the diel vertical migration of copepods under Arctic sea ice during the midnight sun. J. Plankton Res..

[bib24] Giering S.L.C., Sanders R., Lampitt R.S., Anderson T.R., Tamburini C., Boutrif M., Zubkov M.V., Marsay C.M., Henson S.A., Saw K. (2014). Reconciliation of the carbon budget in the ocean’s twilight zone. Nature.

[bib25] Golombek D.A., Rosenstein R.E. (2010). Physiology of circadian entrainment. Physiol. Rev..

[bib26] Gotow T., Nishi T. (2008). Simple photoreceptors in some invertebrates: physiological properties of a new photosensory modality. Brain Res..

[bib27] Häfker N.S., Meyer B., Last K.S., Pond D.W., Hüppe L., Teschke M. (2017). Circadian clock involvement in zooplankton diel vertical migration. Curr. Biol..

[bib28] Häfker N.S., Teschke M., Last K.S., Pond D.W., Hüppe L., Meyer B. (2018). *Calanus finmarchicus* seasonal cycle and diapause in relation to gene expression, physiology, and endogenous clocks. Limnol. Oceanogr..

[bib29] Helm B., Visser M.E., Schwartz W., Kronfeld-Schor N., Gerkema M., Piersma T., Bloch G. (2017). Two sides of a coin: ecological and chronobiological perspectives of timing in the wild. Philos. Trans. R. Soc. B Biol. Sci..

[bib30] Hobbs L., Cottier F.R., Last K.S., Berge J. (2018). Pan-Arctic diel vertical migration during the polar night. Mar. Ecol. Prog. Ser..

[bib31] Huffeldt N.P. (2020). Photic barriers to poleward range-shifts. Trends Ecol. Evol..

[bib32] Hughes M.E., DiTacchio L., Hayes K.R., Vollmers C., Pulivarthy S., Baggs J.E., Panda S., Hogenesch J.B. (2009). Harmonics of circadian gene transcription in mammals. PLoS Genet..

[bib33] Hughes M.E., Abruzzi K.C., Allada R., Anafi R., Arpat A.B., Asher G., Baldi P., de Bekker C., Bell-Pedersen D., Blau J. (2017). Guidelines for genome-scale analysis of biological rhythms. J. Biol. Rhythms.

[bib34] Hüppe L., Payton L., Last K., Wilcockson D., Ershova E., Meyer B. (2020). Evidence for oscillating circadian clock genes in the copepod *Calanus finmarchicus* during the summer solstice in the high Arctic. Biol. Lett..

[bib35] Ibáñez-Tejero L., Ladah L.B., Sánchez-Velasco L., Barton E.D., Filonov A. (2018). Vertical distribution of zooplankton biomass during internal tidal forcing under mesoscale conditions of upwelling and relaxation. Cont. Shelf Res..

[bib36] Lenz P.H., Roncalli V., Hassett R.P., Wu L.-S., Cieslak M.C., Hartline D.K., Christie A.E. (2014). *De novo* assembly of a transcriptome for *Calanus finmarchicus* (Crustacea, Copepoda) – the dominant zooplankter of the North Atlantic Ocean. PLoS One.

[bib37] Li J., Grant G.R., Hogenesch J.B., Hughes M.E., Sehgal A. (2015). Chapter Sixteen - considerations for RNA-seq analysis of circadian rhythms. Methods in Enzymology.

[bib38] Li J., Grant G.R., Hogenesch J.B., Hughes M.E. (2015). Considerations for RNA-seq analysis of circadian rhythms. Methods Enzymol..

[bib39] Massicotte P., Amiraux R., Amyot M.-P., Archambault P., Ardyna M., Arnaud L., Artigue L., Aubry C., Ayotte P., Bécu G. (2020). Green Edge ice camp campaigns: understanding the processes controlling the under-ice Arctic phytoplankton spring bloom. Earth Syst. Sci. Data.

[bib40] Mat A.M., Sarrazin J., Markov G.V., Apremont V., Dubreuil C., Eché C., Fabioux C., Klopp C., Sarradin P.-M., Tanguy A. (2020). Biological rhythms in the deep-sea hydrothermal mussel *Bathymodiolus azoricus*. Nat. Commun..

[bib41] Mauvoisin D., Dayon L., Gachon F., Kussmann M. (2015). Proteomics and circadian rhythms: it’s all about signaling!. Proteomics.

[bib42] McDonald M.J., Rosbash M. (2001). Microarray analysis and organization of circadian gene expression in *Drosophila*. Cell.

[bib43] Mermet J., Yeung J., Naef F. (2017). Systems chronobiology: global analysis of gene regulation in a 24-Hour periodic world. Cold Spring Harb. Perspect. Biol..

[bib44] Nandi A., Yan L.-J., Jana C.K., Das N. (2019). Role of catalase in oxidative stress- and age-associated degenerative diseases. Oxid. Med. Cell. Longev..

[bib45] O’Neill J.S., Lee K.D., Zhang L., Feeney K., Webster S.G., Blades M.J., Kyriacou C.P., Hastings M.H., Wilcockson D.C. (2015). Metabolic molecular markers of the tidal clock in the marine crustacean *Eurydice pulchra*. Curr. Biol..

[bib46] Oziel L., Massicotte P., Randelhoff A., Ferland J., Vladoiu A., Lacour L., Galindo V., Lambert-Girard S., Dumont D., Cuypers Y. (2019). Environmental factors influencing the seasonal dynamics of spring algal blooms in and beneath sea ice in western Baffin Bay. Elem. Sci. Anth..

[bib47] Patton D.F., Mistlberger R.E. (2013). Circadian adaptations to meal timing: neuroendocrine mechanisms. Front. Neurosci..

[bib48] Payton L., Perrigault M., Hoede C., Massabuau J.-C., Sow M., Huvet A., Boullot F., Fabioux C., Hegaret H., Tran D. (2017). Remodeling of the cycling transcriptome of the oyster *Crassostrea gigas* by the harmful algae *Alexandrium minutum*. Sci. Rep..

[bib49] Payton L., Noirot C., Hoede C., Hüppe L., Last K., Wilcockson D., Ershova E.A., Valière S., Meyer B. (2020). Daily transcriptomes of the copepod *Calanus finmarchicus* during the summer solstice at high Arctic latitudes. Sci. Data.

[bib50] Petrusevich V.Y., Dmitrenko I.A., Niemi A., Kirillov S.A., Kamula C.M., Kuzyk Z.Z.A., Barber D.G., Ehn J.K. (2020). Impact of tidal dynamics on diel vertical migration of zooplankton in Hudson Bay. Ocean Sci..

[bib51] Pittà C.D., Biscontin A., Albiero A., Sales G., Millino C., Mazzotta G.M., Bertolucci C., Costa R. (2013). The Antarctic krill *Euphausia superba* shows diurnal cycles of transcription under natural conditions. PLoS One.

[bib52] Putker M., O’Neill J.S. (2016). Reciprocal control of the circadian clock and cellular redox state - a critical appraisal. Mol. Cells.

[bib53] Reygondeau G., Beaugrand G. (2011). Future climate-driven shifts in distribution of *Calanus finmarchicus*. Glob. Change Biol..

[bib54] Saikkonen K., Taulavuori K., Hyvönen T., Gundel P.E., Hamilton C.E., Vänninen I., Nissinen A., Helander M. (2012). Climate change-driven species’ range shifts filtered by photoperiodism. Nat. Clim. Change.

[bib55] Sanders R., Henson S.A., Koski M., De La Rocha C.L., Painter S.C., Poulton A.J., Riley J., Salihoglu B., Visser A., Yool A. (2014). The biological carbon pump in the North Atlantic. Prog. Oceanogr..

[bib56] Satoh A., Terai Y. (2019). Circatidal gene expression in the mangrove cricket Apteronemobius asahinai. Sci. Rep..

[bib57] Schmal C., Herzel H., Myung J. (2020). Clocks in the wild: entrainment to natural light. Front. Physiol..

[bib58] Schmitt F.G., Devreker D., Dur G., Souissi S. (2011). Direct evidence of tidally oriented behavior of the copepod *Eurytemora affinis* in the Seine estuary. Ecol. Res..

[bib59] Schnytzer Y., Simon-Blecher N., Li J., Ben-Asher H.W., Salmon-Divon M., Achituv Y., Hughes M.E., Levy O. (2018). Tidal and diel orchestration of behaviour and gene expression in an intertidal mollusc. Sci. Rep..

[bib60] Søreide J.E., Falk-Petersen S., Hegseth E.N., Hop H., Carroll M.L., Hobson K.A., Blachowiak-Samolyk K. (2008). Seasonal feeding strategies of *Calanus* in the high-Arctic Svalbard region. Deep Sea Res. Part Top. Stud. Oceanogr..

[bib61] Søreide J.E., Carroll M.L., Hop H., Jr W.G.A., Hegseth E.N., Falk-Petersen S. (2013). Sympagic-pelagic-benthic coupling in Arctic and Atlantic waters around Svalbard revealed by stable isotopic and fatty acid tracers. Mar. Biol. Res..

[bib62] Tarrant A.M., Nilsson B., Hansen B.W. (2019). Molecular physiology of copepods - from biomarkers to transcriptomes and back again. Comp. Biochem. Physiol. Part D Genomics Proteomics.

[bib63] Tarrant A.M., Helm R.R., Levy O., Rivera H.E. (2019). Environmental entrainment demonstrates natural circadian rhythmicity in the cnidarian *Nematostella vectensis*. J. Exp. Biol..

[bib64] Tessmar-Raible K., Raible F., Arboleda E. (2011). Another place, another timer: marine species and the rhythms of life. BioEssays.

[bib65] Tran D., Perrigault M., Ciret P., Payton L. (2020). Bivalve mollusc circadian clock genes can run at tidal frequency. Proc. R. Soc. B Biol. Sci..

[bib66] Wallace M.I., Cottier F.R., Berge J., Tarling G.A., Griffiths C., Brierley A.S. (2010). Comparison of zooplankton vertical migration in an ice-free and a seasonally ice-covered Arctic fjord: an insight into the influence of sea ice cover on zooplankton behavior. Limnol. Oceanogr..

[bib67] Weng Y., DiRusso C.C., Reilly A.A., Black P.N., Ding X. (2005). Hepatic gene expression changes in mouse models with liver-specific deletion or global suppression of the *NADPH-cytochrome P450 reductase* gene. Mechanistic implications for the regulation of microsomal cytochrome P450 and fatty liver phenotype. J. Biol. Chem..

[bib68] Westermark P.O., Herzel H. (2013). Mechanism for 12 hr rhythm generation by the circadian clock. Cell Rep..

[bib69] Zhu B., Dacso C.C., O’Malley B.W. (2018). Unveiling “musica universalis” of the cell: a brief history of biological 12-hour rhythms. J. Endocr. Soc..

