# Supplementary material for: Widely rhythmic transcriptome in Calanus finmarchicus during the high Arctic summer solstice period
Source: iScience. 2020 Dec 11;24(1):101927. doi: 10.1016/j.isci.2020.101927 (PMC7770977; doi:10.1016/j.isci.2020.101927)
Supplement: Document S1. Transparent Methods and Figures S1–S4 [file mmc1.pdf]

iScience, Volume *24*

Supplemental Information

Widely rhythmic transcriptome

in *Calanus finmarchicus*

during the high Arctic summer solstice period

Laura Payton, Lukas Hüppe, Céline Noirot, Claire Hoede, Kim S. Last, David Wilcockson, Elizaveta Ershova, Sophie Valière, and Bettina Meyer

Transparent Methods

EXPERIMENTAL MODEL AND SUBJECTS DETAILS

All animal work was conducted in accordance with local legislation. All investigations were performed on CV life stages of the copepod *Calanus finmarchicus* (Gunnerus, 1770). Copepods were sorted for species (*C. finmarchicus*) and stage (CV stage copepodites) at 2˚C under a stereo microscope using morphological characteristics. To distinguish *C. finmarchicus* from its closely related Arctic congener *C. glacialis*, the redness of the antenna, which has been shown to be a good indicator in the regions, was particularly used (Nielsen et al., 2014). Morphological identification method was validated by molecular species identification on a subset of samples from the same stations. DNA was extracted from individual copepods using the HotShot method (Truett et al., 2000), and the species-specific nuclear insertion/deletion (InDel) marker G-150 was amplified using a modified protocol from Smolina et al. (2014). Identification was done by accessing the size of the resulting amplicon via electrophoresis on a 2% agarose gel. 99 % of the individuals identified as *C. finmarchicus* by the morphological identification method were also identified as *C. finmarchicus* by the molecular identification method (n=305 individuals).

METHOD DETAILS

Study sites characteristics

Sampling was conducted during Cruise JR17006 of the *RRS James Clark Ross* in summer 2018 at two stations along a latitudinal gradient. The *South* station was located in the southern Barents Sea (B13; 74.5 °N, 30 °E) and the *North* station in the Nansen Basin (JR85; 82.56 °N, 30.85 °E) (Figure 1). Water depth at *South* was 360 m and at *North* was 3700 m. During sampling times, the ice edge was located at about 81° to 82° N, roughly following the shelf slope north of Svalbard, thus *South* station (74.5 °N) was ice-free, whereas *North* station (82.56 °N) was located within the ice cover. The sun's altitude was always above the horizon but still showed diel oscillations of altitude above the horizon from 7.7 ° at midnight to 38.6 ° at midday at *South*, and from 16 ° at midnight to 30.9 ° at midday at *North*, at the times of sampling (local time, UTC +2). Sites were exposed to semidiurnal tide regimes, i.e., 2 tidal cycles per day, with a maximum amplitude of ± 0.36 m at *South* and ± 0.47 m at *North* at times of sampling. Atmospheric PAR measurements and additional physical characteristics of the sampling sites are available in the publication of Hüppe et al. (2020).

Field sampling time series

The sampling strategy was specifically designed for the detection of rhythmic transcripts. Sampling covered a complete 24h cycle at 4h intervals, resulting in seven time points per station. At each station, sampling was performed in similar times frames: 14-15h, 18-19h, 22-23h, 2-3h, 6-7h, and 10-11h (all times noted in local time (UTC+2)). Sampling at *South* station started on 30th June (9 days after the summer solstice) at 14-15h and ended on 1st July at 14-15h. Sampling at *North* station started on 18th June (3 days before the summer solstice) at 10-11h and ended on 19th June at 10-11h. Field sampling interval was conducted on a near semi-lunar cycle (12 days apart) to ensure tidal and solar cycles were in phase. At each timepoint the water column was sampled from 200 m to the surface with vertical hauls of a WP2 plankton net (opening ⌀: 57 cm, net length: 236 cm, mesh size: 200 µm) with a meshed bucket cod end (mesh size: 200 µm) at a speed of 0.5 m*s-1. Transferring the animals from the net into the stabilization solution was done within less than 12

minutes for all samplings. A 12h period of incubation at 2 - 4˚C was allowed to soak the samples thoroughly with the RNAlater stabilization solution (Ambion, UK) before they were transferred to -80˚C for further transport and storage.

RNA extraction

Copepods were sorted at 2˚C and for each timepoint and station, 3 replicates of 15 *C. finmachicus* CV were analyzed (315 individuals per station in total). Each replicate was homogenized in 600 µl of TRIzol® reagent (ThermoFisher Scientific, USA) with a Precellys® 24 Tissue Homogenizer (Bertin Instruments, France). For RNA extraction, a Phenol/Chloroform based single-step extraction in combination with a spin column based solid phase extraction (Direct-zol™ RNA MiniPrep Kit, Zymo Research, USA) was used. Genomic DNA was removed by DNase I digestion on column as part of the RNA extraction kit and total RNA was eluted in ultra-pure water. Total RNA samples were stored at -80°C and send to GeT-PlaGe core facility on dry ice. RNA purity and quantity was checked on a NanoDrop 8000 spectrophotometer (ThermoFisher Scientific, USA) and RNA integrity was checked using a Fragment Analyzer (Advanced Analytical Technologies, Inc., Iowa, USA; RNA Kit (15nt) Standard Sensitivity, Agilent).

RNA sequencing

RNAseq was performed at the GeT-PlaGe core facility, INRA Toulouse. The 42 RNA-seq libraries were prepared according to Illumina’s protocols using the Illumina TruSeq Stranded mRNA sample prep kit to analyze mRNA. Briefly, mRNA were selected using poly-T beads. Then, RNA were fragmented to generate double stranded cDNA and adaptors were ligated to be sequenced. 11 cycles of PCR were applied to amplify libraries. Library quality was assessed using a Fragment Analyser (Advanced Analytical Technologies, Inc., Iowa, USA) and libraries were quantified by qPCR using the Kapa Library Quantification Kit (Roche). RNA-seq experiments have been performed on a NovaSeq S4 lane (Illumina, California, USA) using a paired-end read length of 2x150 pb with the Illumina NovaSeq Reagent Kits.

RNA sequencing bioinformatics analysis

Details on bioinformatics analysis are available in the article by Payton et al., 2020. Sequenced reads were aligned by BWA MEM (http://bio-bwa.sourceforge.net/bwa.shtml) to the reference *de novo* transcriptome of *Calanus finmarchicus* (Lenz et al., 2014) and quantification matrix was generate thanks to samtools idxstat (Li et al., 2009) results. The percentage of mapped (single) reads is 95.5 ± 0.001 %, and the percentage of multimapped reads is 2.6 ± 0.05 %, attested for a good alignment of the dataset on the reference transcriptome. The 76 550 transcripts with more than 1 cpm were selected over the 206 012 transcripts for further analysis. The reference transcriptome was annotated with diamond v0.9.22 (Buchfink et al., 2015) against Swissprot, Trembl and NR. Only the best hits of each database were selected if i) the percent of the query length covered by the alignment was higher than 60% ; ii) the percent of the subject length covered by the alignment was higher than 40%; iii) the percent of identity of the alignment was higher than 40%. Interproscan v5.29-68.0 (Jones et al., 2014) was used to associate a Gene Ontology to contigs. The mapped reads were down-sampled to the lowest number of mapped reads among the 42 samples (down-sampling normalization) with StreamSampler.jar (https://github.com/shenkers/sampling), i.e. to 70.4 million reads per sample for all samples (Hughes et al., 2017; Koike et al., 2012; Li et al., 2015), in order to adjust for differences in sequencing depth among samples. Finally, 76 550 transcripts per station were analyzed for statistical rhythmic analysis and Gene Ontology study. Row transcriptomic data, annotation table and down-sampling normalization table are available in the NCBI Bioproject PRJNA628886 (https://www.ncbi.nlm.nih.gov/bioproject/PRJNA628886) and in the figshare collection 5127704 (https://doi.org/10.6084/m9.figshare.c.5127704).

Real-time quantitative PCR verification

RNA sequencing results were verified through RT-qPCR analysis (Figure S1). Raw data of expression from Hüppe et al. (2020) of six circadian core clock genes (*clock*, *cycle*, *period1*, *timeless*, *cryptochrome2*, *vrille*) and 2 circadian clock-related genes (*cryptochrome1* and *doubletime2*), relative to the geometric mean of the most stable reference genes (*elongation factor 1-*α and *16s rRNA*), were analyzed on the exact same samples analyzed in this study. In parallel, these clock and clock-related transcripts were identified in the

transcriptomic results based on Christie et al. (2013). Profiles of expression were plotted and statistical rhythmic analysis were performed on both analyzes (RNA sequencing and RT-qPCR) and showed strong similarities (Figure S1), comforting the relevance of the rhythmic transcriptomic analysis.

QUANTIFICATION AND STATISTICAL ANALYSIS

Environmental parameters

Information on the location of the sea ice edge at the time of sampling at *North* were obtained from ice concentration maps available from the *meereisportal* (Grosfeld et al., 2016). Modeled data of sun altitude were obtained from the United States Naval Observatory (https://aa.usno.navy.mil/data/docs/AltAz.php, USNO, USA) Information on the tidal dynamics have been drawn from the TPX08 model (Egbert and Erofeeva, 2002) by using the OTPS package (Tidal Prediction Software, http://www-po.coas.oregonstate.edu/~poa/www-po/research/po/research/tide/index.html), via the mbotps program (MB-System; Caress and Chayes, 2016).

Rhythmic analysis

Rhythmic analysis of transcripts over the 24h cycles was performed in RStudio (Version 1.2.1335, R version 3.6.3, R Core Team, 2013), using RAIN package. RAIN was specifically designed to detect rhythms in biological datasets independent of waveform by using a non-parametric approach (Thaben and Westermark, 2014). The 76 550 transcripts of each station were tested together (153 100 total transcripts). The false discovery rate of the *p-values* was corrected using the Benjamini-Hochberg method (Benjamini and Hochberg, 1995). The time series have been tested using the three samples per timepoint as replicates and the "independent" mode. Based on the sampling plan and RAIN algorithm terms of use, the following periods were tested: 24h, 20h, 16h and 12h. Period lengths of 20h and 24 h were in the circadian range, while period lengths of 12 and 16h were in the ultradian range. In this study, gene expression oscillating in a circadian or ultradian period range will be called "daily" and "ultradian", respectively, assuming that they are the results of an endogenous clock regulation or a direct response to environmental factors (Helm et al., 2017). For each

period and each transcript, the waveform yielding the most significant result was selected. Then, for each transcript, the period yielding the most significant result was selected. The associated phase was determined by RAIN algorithm. When the period length was shorter than the time frame (24h), the second and third phases were deducted according to the RAIN model. Heatmaps were plotted in RStudio using heatmap3 package (https://www.rdocumentation.org/packages/heatmap3). Relative levels of expression were calculated for each transcripts by normalizing levels of expression to the median of the seven time points. For visualization purposes, relative levels of expression above 1 were binned to 1. For each heatmaps, transcripts were ordered by phases. Amplitudes of oscillating transcripts were calculated as ((maximum value - minimum value) / minimum value) (Hughes et al., 2012; Payton et al., 2017). Normalization by the minimum value makes it possible to be exempt from the differences in the level of expression of each transcript. For the RT-qPCR verification of RNA sequencing (6 core circadian clock genes and 2 circadian clock-related genes, Figure S1), the period yielding the most significant result in each period range (circadian and ultradian) was selected, thus assuming two hypothetical significant period lengths per transcripts.

Gene Ontology

Gene Ontology (GO) analysis of biological processes were performed in RStudio (Version 1.2.1335, R version 3.6.3, R Core Team, 2013), using the topGO package (Rahnenfuhrer J, 2019). Enrichment analyses were performed using Fisher's exact test and *Weight* method (Alexa et al., 2006) thanks to homemade scripts (https://forgemia.inra.fr/bios4biol/bioinfo-utils/-/blob/master/bin/GOEnrichment.R). The false discovery rate of the *p*-values was corrected using the Benjamini-Hochberg method (Benjamini and Hochberg, 1995; McDonald, 2014). For the enrichment analyses presented Fig. S2B, S3B and S4B, circadian and ultradian transcripts were tested together.

REFERENCES

Alexa, A., Rahnenführer, J., and Lengauer, T. (2006). Improved scoring of functional groups from gene expression data by decorrelating GO graph structure. Bioinformatics *22*, 1600–1607.

Benjamini, Y., and Hochberg, Y. (1995). Controlling the False Discovery Rate: A Practical and Powerful Approach to Multiple Testing. J. R. Stat. Soc. Ser. B Methodol. *57*, 289–300.

Buchfink, B., Xie, C., and Huson, D.H. (2015). Fast and sensitive protein alignment using DIAMOND. Nat. Methods *12*, 59–60.

Caress, D.W., and Chayes, D., N. (2016). MB-System Version 5.5.2284. Open source software distributed from the MBARI and L-DEO web sites.

Christie, A.E., Fontanilla, T.M., Nesbit, K.T., and Lenz, P.H. (2013). Prediction of the protein components of a putative *Calanus finmarchicus* (Crustacea, Copepoda) circadian signaling system using a de novo assembled transcriptome. Comp. Biochem. Physiol. Part D Genomics Proteomics *8*, 165–193.

Egbert, G.D., and Erofeeva, S.Y. (2002). Efficient Inverse Modeling of Barotropic Ocean Tides. J. Atmospheric Ocean. Technol. *19*, 183–204.

Grosfeld, K., Treffeisen, R., Asseng, J., Bartsch, A., Bräuer, B., Fritzsch, B., Gerdes, R., Hendricks, S., Hiller, W., Heygster, G., et al. (2016). Online Sea-Ice Knowledge and Data Platform.

Helm, B., Visser, M.E., Schwartz, W., Kronfeld-Schor, N., Gerkema, M., Piersma, T., and Bloch, G. (2017). Two sides of a coin: ecological and chronobiological perspectives of timing in the wild. Philos. Trans. R. Soc. B Biol. Sci. *372*, 20160246.

Hughes, M.E., Grant, G.R., Paquin, C., Qian, J., and Nitabach, M.N. (2012). Deep sequencing the circadian and diurnal transcriptome of Drosophila brain. Genome Res. 22, 1266–1281.

Hughes, M.E., Abruzzi, K.C., Allada, R., Anafi, R., Arpat, A.B., Asher, G., Baldi, P., de Bekker, C., Bell-Pedersen, D., Blau, J., et al. (2017). Guidelines for Genome-Scale Analysis of Biological Rhythms. J. Biol. Rhythms *32*, 380–393.

Hüppe, L., Payton, L., Last, K., Wilcockson, D., Ershova, E., and Meyer, B. (2020). Evidence for oscillating circadian clock genes in the copepod *Calanus finmarchicus* during the summer solstice in the high Arctic. Biol. Lett. *16*, 20200257.

Jones, P., Binns, D., Chang, H.-Y., Fraser, M., Li, W., McAnulla, C., McWilliam, H., Maslen, J., Mitchell, A., Nuka, G., et al. (2014). InterProScan 5: genome-scale protein function classification. Bioinformatics *30*, 1236–1240.

Koike, N., Yoo, S.-H., Huang, H.-C., Kumar, V., Lee, C., Kim, T.-K., and Takahashi, J.S. (2012). Transcriptional Architecture and Chromatin Landscape of the Core Circadian Clock in Mammals. Science *338*, 349–354.

Lenz, P.H., Roncalli, V., Hassett, R.P., Wu, L.-S., Cieslak, M.C., Hartline, D.K., and Christie, A.E. (2014). *De Novo* Assembly of a Transcriptome for *Calanus finmarchicus* (Crustacea, Copepoda) – The Dominant Zooplankter of the North Atlantic Ocean. PLOS ONE *9*, e88589.

Li, H., Handsaker, B., Wysoker, A., Fennell, T., Ruan, J., Homer, N., Marth, G., Abecasis, G., and Durbin, R. (2009). The Sequence Alignment/Map format and SAMtools. Bioinformatics *25*, 2078–2079.

Li, J., Grant, G.R., Hogenesch, J.B., and Hughes, M.E. (2015). Considerations for RNA-seq analysis of circadian rhythms. Methods Enzymol. *551*, 349–367.

McDonald, J.H. (2014). Multiple comparisons. In Handbook of Biological Statistics (3rd Ed.)., (Sparky House Publishing, Baltimore, Maryland.), pp. 254–260.

Nielsen, T.G., Kjellerup, S., Smolina, I., Hoarau, G., and Lindeque, P. (2014). Live discrimination of *Calanus glacialis* and *C. finmarchicus* females: can we trust phenological differences? Mar. Biol. *161*, 1299–1306.

Payton, L., Perrigault, M., Hoede, C., Massabuau, J.-C., Sow, M., Huvet, A., Boullot, F., Fabioux, C., Hegaret, H., and Tran, D. (2017). Remodeling of the cycling transcriptome of the oyster *Crassostrea gigas* by the harmful algae *Alexandrium minutum*. Sci. Rep. 7, 3480.

Payton, L., Noirot, C., Hoede, C., Hüppe, L., Last, K., Wilcockson, D., Ershova, E.A., Valière, S., and Meyer, B. (2020). Daily transcriptomes of the copepod *Calanus finmarchicus* during the summer solstice at high Arctic latitudes. Sci. Data 7, 415.

R Core Team (2013). R: The R project for statistical computing.

Rahnenfuhrer J, A.A. (2019). topGO: Enrichment Analysis for Gene Ontology.

Smolina, I., Kollias, S., Poortvliet, M., Nielsen, T.G., Lindeque, P., Castellani, C., Møller, E.F., Blanco-Bercial, L., and Hoarau, G. (2014). Genome- and transcriptome-assisted development of nuclear insertion/deletion markers for *Calanus* species (Copepoda: Calanoida) identification. Mol. Ecol. Resour. *14*, 1072–1079.

Thaben, P.F., and Westermark, P.O. (2014). Detecting rhythms in time series with RAIN. J. Biol. Rhythms *29*, 391–400.

Truett, G.E., Heeger, P., Mynatt, R.L., Truett, A.A., Walker, J.A., and Warman, M.L. (2000). Preparation of PCR-quality mouse genomic DNA with hot sodium hydroxide and tris (HotSHOT). BioTechniques *29*, 52, 54.

Supplemental figures

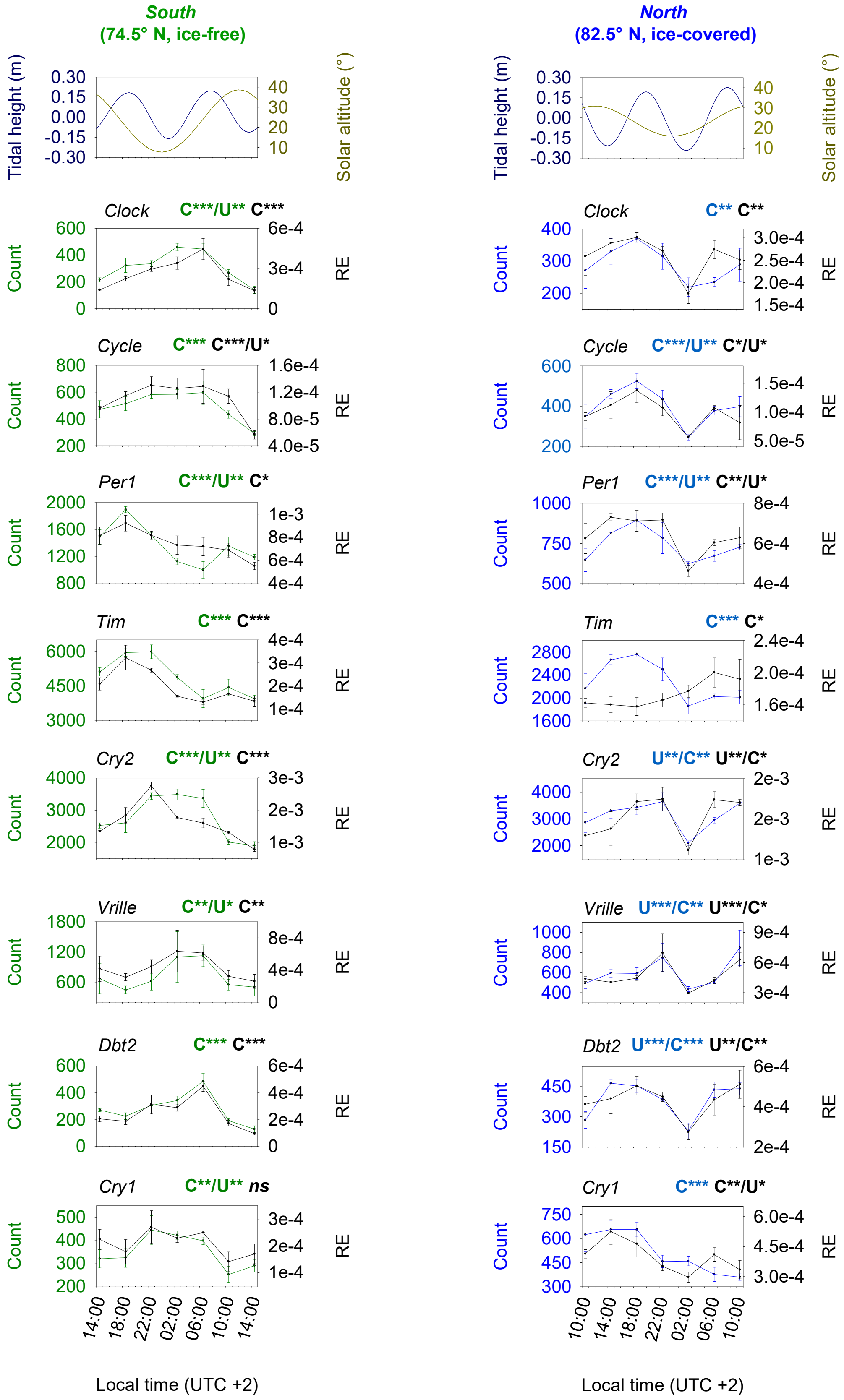

Figure S1: Real-time quantitative PCR validation of RNA sequencing analysis with 6 core circadian clock and 2 clock-related genes at each station, related to Figures 1 and 2. Data from RNA-seq (Count, green and blue) and from RT-qPCR (RE, relative expression, black) are compared at each station. Significant levels of oscillations detected by RAIN (Benjamini-Hochberg adjusted *p*-values) within circadian (C, 20/24 h) and ultradian (U, 12/16 h) period ranges were indicated with stars: * Adjusted-*p* ≤0.05, ** Adjusted-*p* <0.01, *** Adjusted-*p* <0.001.

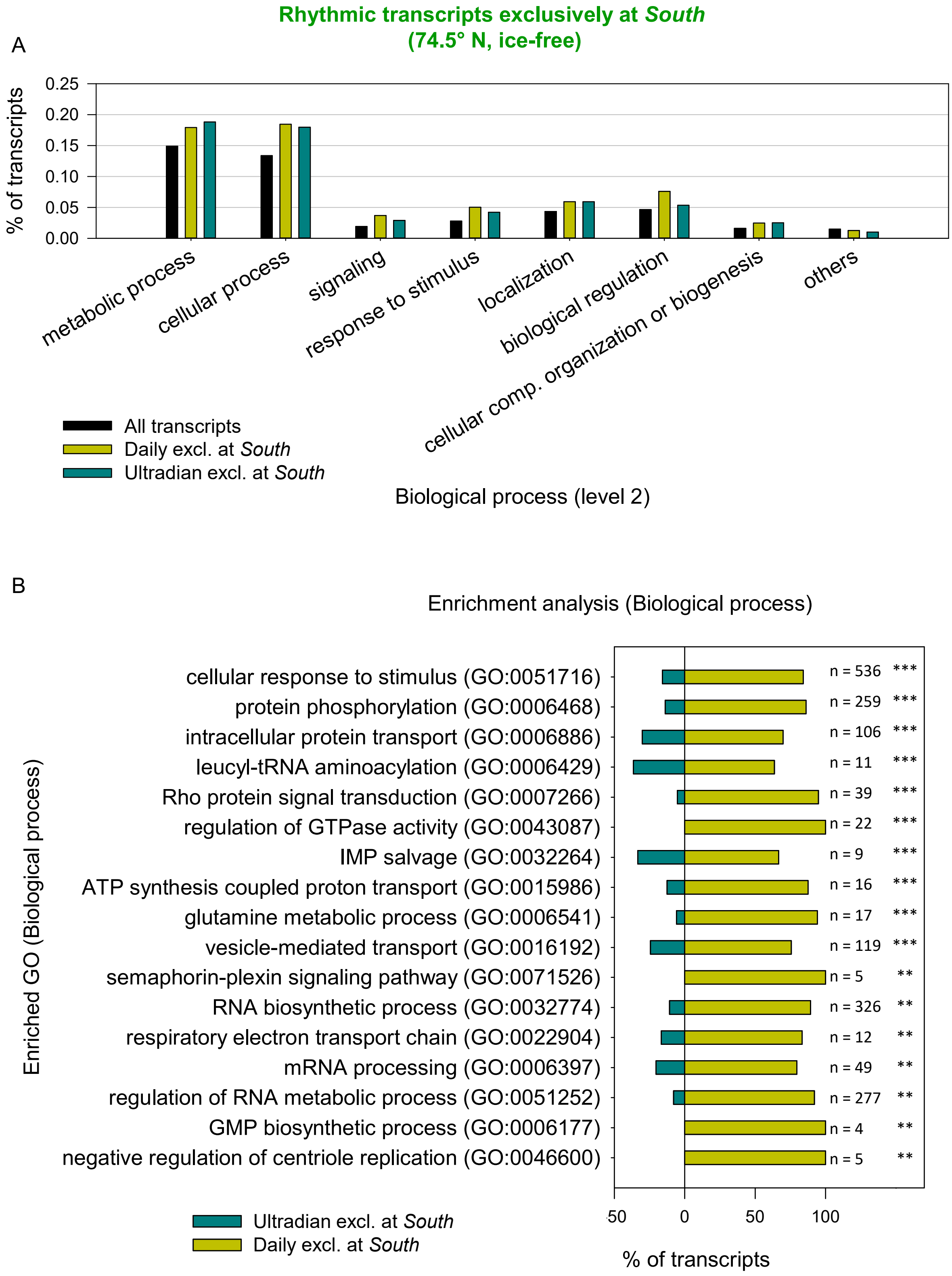

Figure S2: GO analysis of rhythmic transcripts exclusively at *South* (74.5°N, ice-free), related to Figure 3. See legend next page.

Legend Figure S2: GO analysis of rhythmic transcripts exclusively at *South* (74.5°N, ice-free), related to Figure 3. A. Distribution of GO at level 2 (Biological process) in all transcripts (rhythmic and non rhythmic, n = 76550), daily transcripts exclusively at *South* (n = 9859), and ultradian transcripts exclusively at *South* (n = 2242) (significant rhythmicity with an adjusted p-value < 0.001). Distribution of metabolic process (GO:0008152), cellular process (GO:0009987), signaling (GO:0023052), multicellular organismal process (GO:0032501), developmental process (GO:0032502), response to stimulus (GO:0050896), localization (GO:0051179), biological regulation (GO:0065007), cellular component organization or biogenesis (GO:0071840) and others was expressed in percentage of transcripts per group. B. Enrichment analysis (Biological process) of all rhythmic transcripts exclusively at *South* (74.5°N, ice-free). For each enriched functions (adjusted p-value > 0.01), the percentage of ultradian and daily transcripts in each enriched function were detailed. On the right, "n" indicated the total number of transcripts per enriched function (ultradian and daily), and the stars indicated the level of significance of the enrichment analysis: ** Adjusted-p <0.01, *** Adjusted-p <0.001.

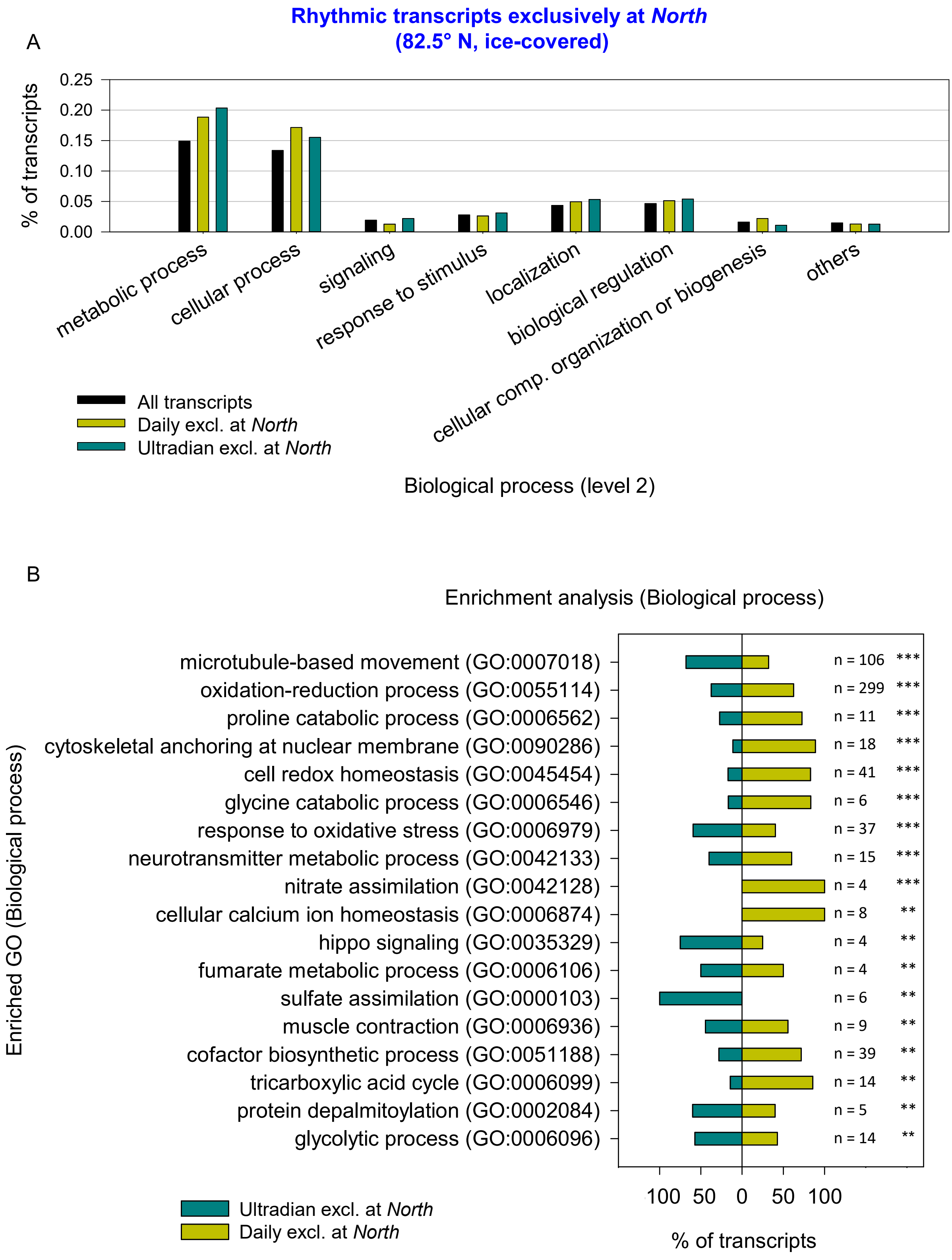

Figure S3: GO analysis of rhythmic transcripts exclusively at *North* (82.5°N, ice-covered), related to Figure 3. See legend next page.

Legend Figure S3: GO analysis of rhythmic transcripts exclusively at *North* (82.5°N, ice-covered), related to Figure 3. A. Distribution of GO at level 2 (Biological process) in all transcripts (rhythmic and non rhythmic, n = 76550), daily transcripts exclusively at *North* (n = 3788), and ultradian transcripts exclusively at *North* (n = 2902) (significant rhythmicity with an adjusted p-value < 0.001). Distribution of metabolic process (GO:0008152), cellular process (GO:0009987), signaling (GO:0023052), multicellular organismal process (GO:0032501), developmental process (GO:0032502), response to stimulus (GO:0050896), localization (GO:0051179), biological regulation (GO:0065007), cellular component organization or biogenesis (GO:0071840) and others was expressed as percentage of transcripts per group. B. Enrichment analysis (Biological process) of all rhythmic transcripts exclusively at *North* (82.5°N, ice-covered). For each enriched functions (adjusted *p*-value > 0.01), the percentage of ultradian and daily transcripts in each enriched function were detailed. On the right, “n” indicated the total number of transcripts per enriched function (ultradian and daily), and the stars indicated the level of significance of the enrichment analysis: ** Adjusted-*p* <0.01, *** Adjusted-*p* <0.001.

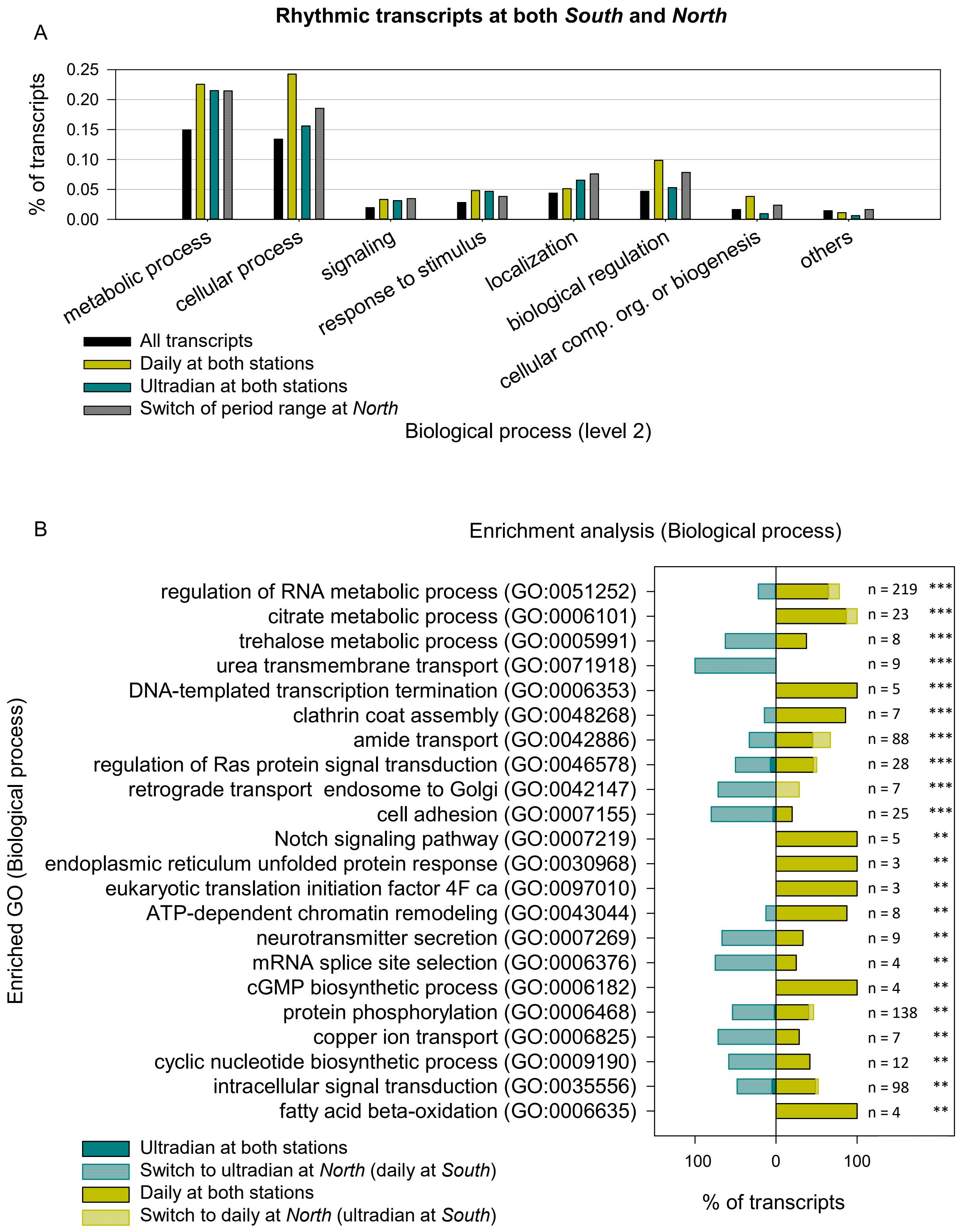

Figure S4: GO analysis of common rhythmic transcripts between *South* (74.5°N, ice-free) and *North* (82.5°N, ice-covered) stations, related to Figure 3. See legend next page.

Legend Figure S4: GO analysis of common rhythmic transcripts between *South* (74.5°N, ice-free) and *North* (82.5°N, ice-covered) stations, related to Figure 3. A. Distribution of GO at level 2 (Biological process) in all transcripts (rhythmic and non rhythmic, n = 76550), daily transcripts at both stations (n = 2814), ultradian transcripts at both stations (n = 322), and rhythmic transcripts at both stations, with a switch of period range at *North* (n = 2808) (significant rhythmicity with an adjusted p-value < 0.001). Distribution of metabolic process (GO:0008152), cellular process (GO:0009987), signaling (GO:0023052), multicellular organismal process (GO:0032501), developmental process (GO:0032502), response to stimulus (GO:0050896), localization (GO:0051179), biological regulation (GO:0065007), cellular component organization or biogenesis (GO:0071840) and others was expressed as percentage of transcripts per group. B. Enrichment analysis (Biological process) of all rhythmic transcripts at both stations. For each enriched functions (adjusted *p*-value > 0.01), the percentage of common ultradian, common daily and common transcripts which change of period range at *North* were detailed. Transcripts which change of period range at *North* were represented as expressed at *North* (opposite period range at *South*). On the right, "n" indicated the total number of transcripts per enriched function (all rhythmicities), and the stars indicated the level of significance of the enrichment analysis: ** Adjusted-*p* <0.01, *** Adjusted-*p* <0.001.
